# In Vitro, Oral Acute, and Repeated 28-Day Oral Dose Toxicity of a Mixed-Valence Polyoxovanadate Cluster

**DOI:** 10.3390/ph16091232

**Published:** 2023-08-30

**Authors:** Mariana de M. Barbosa, Lidiane M. A. de Lima, Widarlane A. da S. Alves, Eucilene K. B. de Lima, Luzia A. da Silva, Thiago D. da Silva, Kahoana Postal, Mohammad Ramadan, Kateryna Kostenkova, Dayane A. Gomes, Giovana G. Nunes, Michelly C. Pereira, Wagner E. da Silva, Mônica F. Belian, Debbie C. Crans, Eduardo C. Lira

**Affiliations:** 1 Centro de Biociências, Departamento de Fisiologia e Farmacologia, Universidade Federal de Pernambuco, Recife 50670-901, PE, Brazil; mariana.melob@ufpe.br (M.d.M.B.); widarlane.angela@ufpe.br (W.A.d.S.A.); kelitabl@gmail.com (E.K.B.d.L.); luzia.abilio@ufrpe.br (L.A.d.S.); thiago.douberin@ufpe.br (T.D.d.S.); dayanejooh@gmail.com (D.A.G.); michelly.pereira@ufpe.br (M.C.P.); 2 Departamento de Química, Universidade Federal Rural de Pernambuco, Recife 52171-900, PE, Brazil; lidianelimaa@gmail.com (L.M.A.d.L.); wesdqr@gmail.com (W.E.d.S.); mfbelian@gmail.com (M.F.B.); 3 Centro Politécnico, Departamento de Química, Universidade Federal do Paraná, Curitiba 81530-900, PR, Brazil; kahoanapostal@gmail.com (K.P.); nunesgg@ufpr.br (G.G.N.); 4 Department of Chemistry, Colorado State University, Fort Collins, CO 80523, USA; mramadan@rams.colostate.edu (M.R.); kostenk@rams.colostate.edu (K.K.); 5 Cell and Molecular Biology Program, Colorado State University, Fort Collins, CO 80523, USA

**Keywords:** polyoxovanadates, pentadecavanadate, mixed valence, toxicology, oral acute toxicity

## Abstract

Polyoxovanadates (POV) are a subgroup of polyoxometalates (POM), which are nanosized clusters with reported biological activities. This manuscript describes the first toxicity evaluation of a mixed-valence polyoxovanadate, pentadecavanadate, (Me_4_N)_6_[V_15_O_36_Cl], abbreviated as V_15_. Cytotoxicity experiments using peripheral blood mononuclear cells (PBMC), larvae of *Artemia salina* Leach, and in vivo oral acute and repeated 28-day doses in mice was carried out. The LC_50_ values in PBMC cells and *A. salina* were 17.5 ± 5.8 μmol L^−1^, and 17.9 µg L^−1^, respectively, which indicates high cytotoxic activity. The toxicity in mice was not observed upon acute exposure in a single dose, however, the V_15_ repeated 28-day oral administration demonstrated high toxicity using 25 mg/kg, 50 mg/kg and, 300 mg/kg doses. The biochemical and hematological analyses during the 28-day administration of V_15_ showed significant alteration of the metabolic parameters related to the kidney and liver, suggesting moderate toxicity. The V_15_ toxicity was attributed to the oxidative stress and lipid peroxidation, once thiobarbituric acid (TBAR) levels significantly increased in both males and females treated with high doses of the POV and also in males treated with a lower dose of the POV. This is the first study reporting a treatment-related mortality in animals acutely administrated with a mixed-valence POV, contrasting with the well-known, less toxic decavanadate. These results document the toxicity of this mixed-valence POV, which may not be suitable for biomedical applications.

## 1. Introduction

Polyoxovanadates (POVs) belong to a subclass of polyanionic molecules made up by group 5 and 6 metal-oxo clusters referred to as polyoxometalates or polyoxidometalates (POM) [1,2,3,4]. Several studies describing POVs have demonstrated anticancer, antimicrobial, and antiviral applications [5,6,7,8,9,10]. Moreover, POVs trigger many biochemical effects such as lipoperoxidation and oxidative stress, affecting metabolic pathways and cell cycle arrest. It interferes with ions transport systems and apoptosis, induces cell morphology changes, inhibits phosphorylases and redox enzymes, and activates or deactivates cell signaling [11,12,13]. Regarding its future clinical use, POV’s toxic effects on various biological systems remain a topic in need of more experimental data. In this work, we followed the guidelines from the Organization of Economic Co-operation and Development (OECD) for measuring the acute oral toxicity and long-term toxicity of chemicals [14]. This study deviates from biological studies showing inhibition of diseased cells or simply measuring inhibition of growth or other biological activities [15]. There are currently few studies in the literature using the OECD guidelines for POVs or other vanadium compounds on healthy cells, which is the focus of this work [16,17,18].

Among POVs, decavanadate ([H_n_V_10_O_28_^(6−n)−^], abbreviated V_10_) is the most commonly investigated POV regarding its chemical, physical, and biological properties. It is found to be stable at acidic pH (pH 3–6) and has shown biological effects, such as inducing oxidative stress processes, stimulating enzymes, and interfering in lipid structures and cellular function [19,20,21,22]. It also has effects as an antidiabetic and anticancer agent [23]. Recently, the transition-metal-monosubstituted decavanadates V_9_Mo and V_9_Pt were found to be effective in inhibiting the growth of Mycobacterium smegmatis [24] and Chinese Hamster Ovary (CHO) cells [12]. In both studies, the V_10_ cluster was reported to be a more effective growth inhibitor; however, in bacteria, the cluster is bound more tightly and hence more cell associated than in the mammalian system, where V_9_Mo and V_9_Pt are readily washed off [12,24].

**Figure 1:**
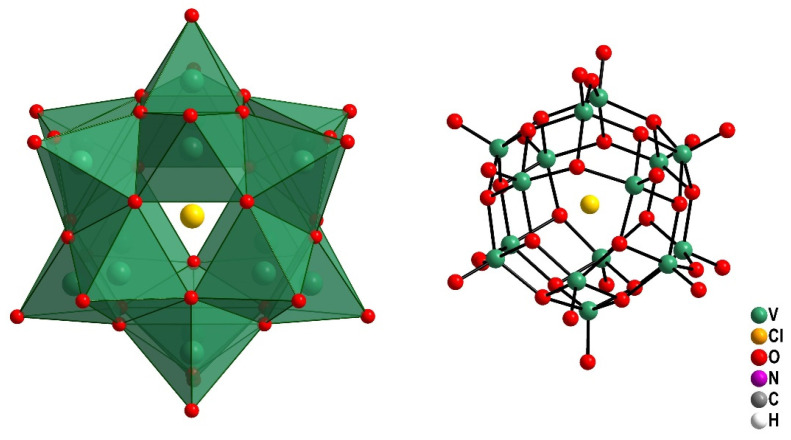
Polyhedral and ball-and-stick representations of [V_15_O_36_Cl]^6−^ (V_15_) anion.

Mixed-valence polyoxovanadates (MVPs) have contributed to an increase in the number of potential therapeutic agents for metabolic diseases and cancer [25,26,27,28]. The MVP [(Me_4_N)_6_[V_15_O_36_Cl] is a polyoxovanadate (abbreviated V_15_, see Figure 1) that contains six Me_4_N^+^ counterions, and the vanadium-oxygen cage of V_15_ consists of one central chlorine atom, eight vanadium(IV) and seven vanadium(V) atoms, and 36 oxygen atoms (Figure 1). V_15_ was effective against the deleterious effect of the deoxyribonucleic acid (DNA) plasmid pUC19 alkylating agents diethyl sulphate and dimethyl sulphate [29]. A chemoprotective effect of 30–40% against diethyl sulphate was additionally shown when using a more complex model of *Escherichia coli* DH5α cell cultures [30]. V_15_ has also demonstrated inhibitory growth effects on microorganisms including *Mycobacterium smegmatis* [31]. Furthermore, the luteinizing hormone receptor (LHR), a G-protein-coupled receptor (GPCR), was used to evaluate V_15_ and other POVs in their interaction with the cell membrane lipid interface of CHO cells [9]. Cell responses for all MVPs were greater than those seen for cells treated with the human chorionic gonadotropin (hCG) hormone, which was used in reference [9]. Despite the promising biological effects described for V_15_, to the best of our knowledge, no evidence of toxicity (in vivo assays) was reported [2,3,29,30,31,32].

Although several POV compounds have demonstrated favorable biological properties, many aspects of the toxicity remain to be further investigated [33]. The toxicological effects of vanadium compounds in biological systems are of great concern and have been well documented in in vitro assays [34,35,36,37]. However, less information on vanadium compounds and POV toxicity is available for in vivo systems, which is critical for any potential biomedical applications [26,27].

Some animal studies have demonstrated that toxicological effects of vanadium compounds are related to the oxidovanadate ion, complex nature, dose, and administration route [35,36,38]. Cellular assays suggest some levels of toxicity are associated with the vanadium compounds due to their commonly observed redox active properties and hence potential for causing oxidative tissue damage, which can be produced by both reactive oxygen species (ROS) and reactive nitrogen species (RNS) [39]. Excess ROS and RNS species result in extensive damage to tissues, nucleic acids, lipids, and proteins through the depletion of sulfhydryl groups, lipids, and reactive parts of biomolecules and their metabolites [39,40].

In this study, toxicological evaluations in both in vitro and in vivo models have been performed to determine any potential human health risk of this multivalent POV cluster, herein abbreviated V_15_. This is the first report of oral single-dose toxicity of a mixed-valence POV in mice.

## 2. Results

### 2.1. Preparation and Characterization of the Pentadecavanadate V_15_

The synthesis of [(CH_3_)_4_N]_6_[V^IV^_8_V^V^_7_O_36_(Cl)] (V_15_) was first described by Muller [41]. More recently, a methodology that uses cheaper and commercially available reactants was developed by Nunes and coworkers [29]. Aiming to produce enough V_15_ to perform in vivo studies, some modifications were made to the previously described procedure to increase scale. Special care must be taken with the control of temperature, because the fresnoite type oxide (NH_4_)_2_VO_3_ is the only product obtained when the reaction takes place at 70 °C [29], and V_15_ can be produced as the major product under reflux.

The material was characterized in both solid and solution states, with the latter studies being performed in aqueous solution and under the conditions of biological studies. Solid-state characterization includes simulated and experimental powder X-ray diffraction studies. The pattern in powder X-ray diffraction experiments of V_15_ was compared to previously reported single-crystal X-ray diffraction structure, showing comparable results (Figure S1, Supplementary Materials). In addition, the FTIR (infrared) spectrum of V_15_ was recorded and showed characteristic bands of the polyoxoanion [42] (Figure S2, Supplementary Materials). The EPR (electronic paramagnetic resonance) spectrum of a pulverized sample of V_15_ was collected at 77 K and found to show the expected broad line centered at 339 mT (g = 1.963 and with Δ_p−p_ of 35 mT, assigned to a ΔMS = ±1 transition (Figure S3, Supplementary Materials). This pattern confirms a partial reduction of the V(V) to V(IV) and is compatible with the intra- and intermolecular exchange interactions between vanadium(IV) centers present in the [V^IV^_8_V^V^_7_O_36_(Cl)]^6−^ polyoxoanion [29,43].

Solution studies included both ^51^V NMR (nuclear magnetic resonance) and EPR studies, as well as UV-Vis spectroscopy. The multivalence of the V_15_ anion affects the ^51^V NMR spectra, since the intact V_15_ is not observed because of the unpaired electrons of the V^IV^-atoms in the cluster. The ^51^V NMR and EPR spectra were recorded in aqueous solutions by dissolving the cluster in water and then adding phosphate-buffered saline (PBS pH 7.4) containing 1.0 mmol L^−1^ CaCl_2_ and 0.50 mmol L^−1^ MgCl_2_. The spectroscopic signature of V_15_ was monitored in a time-dependent study up to 34 h [19] and showed that some V_15_ was hydrolyzed in an aqueous solution and in media to give a mixture of the intact and oxidized V_15_, other oxidovanadium(V) species (H_2_VO_4_^−^, H_2_V_2_O_7_^2−^, V_4_O_12_^4−^, V_5_O_15_^5−^), and the oxidovanadium(IV) complex [VO_2_(OH_2_)_4_]^+^. Other speciation studies were carried out in Luria–Bertani (LB) broth in the presence of other additives at lower pH values (down to 6.0) [44].

The electronic absorption UV-Vis spectra of a 0.025 mmol L^−1^ aqueous solution of V_15_ showed a ligand-to-metal charge transfer (LMCT, p(O)→d(V)) band below 400 nm, and a broad band that extended from 600 nm to the near-infrared was attributed to an intervalence charge transfer transition (IVCT, V^IV^ → V^V^) in the POV anion. The spectrum did not change over a period of 24 h, which is consistent with V_15_ remaining stable in solution (Figure S4, Supplementary Materials).

### 2.2. In Vitro Toxicity

**Figure 2:**
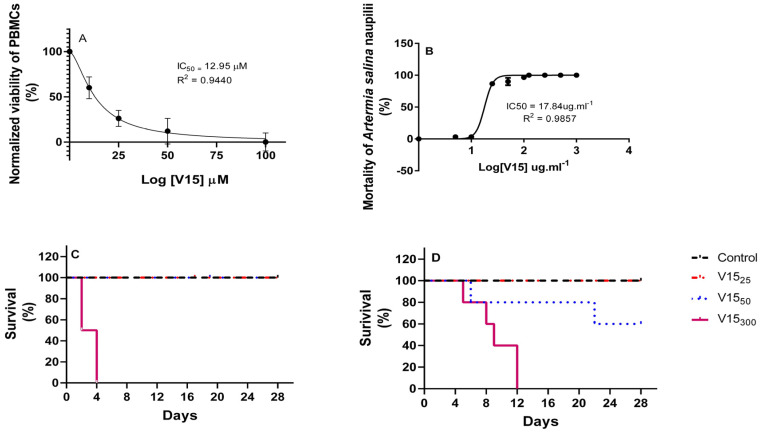
Toxicity effect of the V_15_ on PBMC viability (**A**), *A. saline* nauplii mortality (**B**), female (**C**) and (**D**) male Kaplan–Meier curve of Swiss mice treated with repeated 28-day oral dose. Mean value of triplicate experiments for each countertraction ± SEM (standard error of the mean).

V_15_ with the formula (Me_4_N)_6_[V_15_O_36_Cl] was screened for its cytotoxicity using peripheral blood mononuclear cells (PMBC) using a 3-(4,5-dimethyl-2-thiazolyl)-2,5-diphenyl-2H-tetrazolium bromide (MTT) cytotoxicity assay and a brine shrimp bioassay. The PMBC lethality assay measured in vitro toxicity of V_15_, as shown in Figure 2A at 50 mol·L^−1^. The inhibition stabilized, reaching 50%. In this assay, PBMCs showed decreasing viability at all concentrations tested (Figure 2A). Mean values of viability were 70.43 ± 3.4% at a concentration of 10 µM; 48.61 ± 3.3% at 25 µM; 41.79 ± 5.1% at 50 µM; and 28.06 ± 3.6% at 100 µM, with a dose-dependent reduction in viability. The IC_50_ value of V_15_ was 12.95 µM (10.99–14.91 μmol·L^−1^, R^2^ = 0.9440), indicating a significant inhibition of cell viability (Figure 2A).

The percentage of mortality in the *A. salina* nauplii response increased as the POV concentration increased (Figure 2B). The V_15_ showed a high cytotoxic activity against *A. salina*, with an IC_50_ value of 17.84 (15.75–20.21 μg·mL^−1^, R^2^ = 0.9857), causing 100% mortality in brine shrimp nauplii at a concentration of 125 μg mL^−1^. See below for discussion of the results shown in Figure 2C,D.

### 2.3. Acute Oral Toxicity of V_15_

In this study, the potential for mortality by acute treatment with a POV in animals was evaluated. The animals were examined for abnormal clinical signs and macroscopic findings, all of which were not observed. V_15_ induced a transitory behavioral alteration such as piloerection and lethargy immediately after dosing in the treated animals. The body weight gain, food, and organ mass were not adversely affected by a single oral V_15_ dose. As shown in Table 1, minor differences were observed, and the fluid intake increased by 20% (*p* < 0.05) in the V_15_-treated mice at 300 and 2000 mg/kg treatment levels. The lung weight increased by 25% (*p* < 0.05) for the 300 and 2000 mg/kg levels in the V_15_-treated groups, and liver weight increased by ~10% (*p* < 0.05) only at a higher treatment dose compared to the untreated control group (Table 2). The biochemical analysis of the biological assay is shown in Table 3. V_15_ treatment caused an increase in the aspartate aminotransferase (AST) by 50% (*p* < 0.05) in the 50 mg/kg group and at 2.0-fold (*p* < 0.05) in the 300 and 2000 mg/kg groups. The alanine aminotransferase (ALT) levels were increased 2.5-fold (*p* < 0.05) for the 300 and 2000 mg/kg groups compared to untreated mice. On the other hand, V_15_ reduced albumin content by ~25% (*p* < 0.05) in mice treated with higher doses of V_15_ compared with the control group, without any alteration in blood urea nitrogen (BUN) and total protein levels.

The V_15_ cluster has an acute oral lethal dose (LD_50_) for female mice estimated above the 2000 mg/kg body weight based on an analysis of the dose–mortality relationship. Thus, this POV has a low order of acute toxicity by oral administration in mice, making it a category 5 of acute toxicity material [45].

### 2.4. Repeated-Dose 28-Day Oral Toxicity Study in Rodents

**Figure 3:**
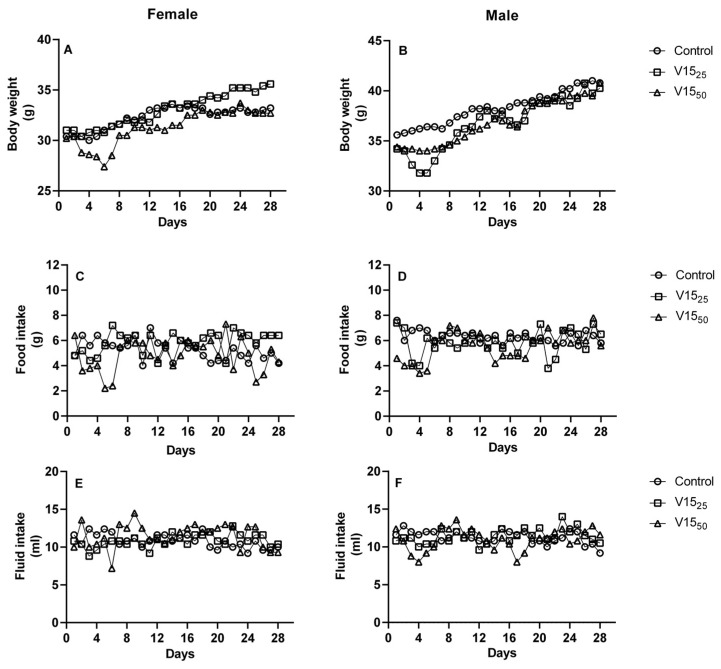
Effect of the 28-day repeated-dose treatment with V_15_ on body weight, food intake, and fluid intake in female (**A**,**C**,**E**) and male (**B**,**D**,**F**) mice. Mean values are presented as mean ± SEM. Mean value with different superscript letters is statistically different at *p* < 0.05 and was analyzed using a one-way ANOVA.

The effects of repeated doses were studied during the 28-day treatment. During the entire experimental period, mice were placed under observation, and signs of toxicity were recorded. No treatment-related changes in the external appearance or general condition were observed in either male or female mice treated with a 25 or a 50 mg/kg b.w. daily dose. The results are displayed in a Kaplan–Meier curve of both Swiss female (Figure 2C) and male mice (Figure 2D). All animals dosed at 300 mg/kg of V_15_ did not die until the 12th day of the treatment. Furthermore, 20% of female mice did not die at the 50 mg/kg level of V_15_ until the 22nd day (Figure 2C). At the high dose, the female mice only survived for 4 days. The survival for the female mice was 100% until the mice were treated with 100 μmol L^−1^; the survival for the male mice was 100% until the mice were treated with 50 or 100 μmol L^−1^, at which point the survival rate was 20 or 8 days. The most common symptoms were diarrhea, lethargy, and weight loss. Body weight and fluid intake of all mice treated with 25 and 50 mg/kg of V_15_ did not show any alteration compared to the untreated control group (Figure 3). However, male and female mice treated with 50 mg/kg had fluctuations in food intake during dosing (Figure 3), but the total food intake in this group was slightly less than that of the V_15–25_ and control groups (Table 4).

The necropsy did not reveal any gross pathological changes compared to the corresponding control groups. In addition, male mice did not show any effects on relative organ weight after V_15_ treatment, except for a slight reduction in teste (20%, *p* < 0.05) and kidney (15%, *p* < 0.05) mass at 50 mg/kg. In female mice, a reduction in tibial (~40%, *p* < 0.05) and reproductive organ mass (uterus and ovaries ~30%, *p* < 0.05) was observed at both doses evaluated (Table 5).

Hematological values of mice treated orally with V_15_ for 28 days are shown in Table 6. In this study, a significant alteration was observed in some hematimetric indices in both male and female mice. V_15_ treatment reduced hemoglobin (~15%, *p* < 0.05), MCV (~20%, *p* < 0.05), and HCT in a dose-dependent manner (25 and 40%, respectively) and stimulated platelet content in a dose-dependent manner (~45 and ~80%, respectively) in treated male mice groups compared to untreated mice. In female mice treated with V_15_ daily for 28 days, a reduction in hemoglobin (~20%, *p* < 0.05), MCV (~15%, *p* < 0.05), and MCH (~15%, *p* < 0.05) was observed after the treatment with both doses, and a reduction in HCT (~70%, *p* < 0.05) was detected only at a higher treatment dose. V_15_ treatment also stimulated platelet content in a dose-dependent manner (~1.5 and ~2-fold, respectively). In addition, V_15_ reduced the WBC (~50%, *p* < 0.05) at a higher dose, and it increased monocytes as the dose increased compared to untreated female mice (Table 6). For other hematological parameters, no significant differences were detected between treated male mice and the control group.

The data shown in Table 7 indicate that V_15_ caused an increase in AST levels in male (~30%, *p* < 0.05) and female (95%, *p* < 0.05) mice. Creatinine was increased only in male mice treated with 50 mg/kg of V_15_ (2.5-fold *p* < 0.05), whereas uric acid levels increased in both male and female mice treated with 25 mg/kg of V_15_ (50%, *p* < 0.05) and male (70%, *p* < 0.05) and female (~80%, *p* < 0.05) mice groups treated with 50 mg/kg of V_15_ compared with the untreated control group. Considering the lipid profile, V_15_ reduced triglyceride (TG) levels in male mice at a higher V_15_ treatment dose (~20%, *p* < 0.05), and there was a reduction in total cholesterol (TC, ~30%, *p* < 0.05) and TG (~20%, *p* < 0.05) in treated female mice compared with the control group.

### 2.5. Oxidative Stress

The oxidative stress parameters evaluated were reduced glutathione (GSH), oxidized glutathione (pGSSG), malondialdehyde (MDA), superoxide dismutase (SOD), and catalase (CAT). The effect of the V_15_ compound on the generation of ROS species in the liver of male and female mice treated for 28 consecutive days is shown in Table 8. In male mice, the V_15_ treatment increased hepatic MDA content, and the SOD activity decreased (~30%, *p* < 0.05). On the other hand, in V_15_-treated female mice, the GSSG levels increased, and the GSH/GSSG ratio decreased, which is consistent with oxidative damage. In addition, at elevated MDA content at a higher treatment dose (~30%, *p* < 0.05), the SOD and CAT activities in female mice were increased compared with the untreated group. The V_15_ treatment clearly impacted female and male mice differently, although evidence of oxidative stress in both male and female mice was apparent.

**Figure 4:**
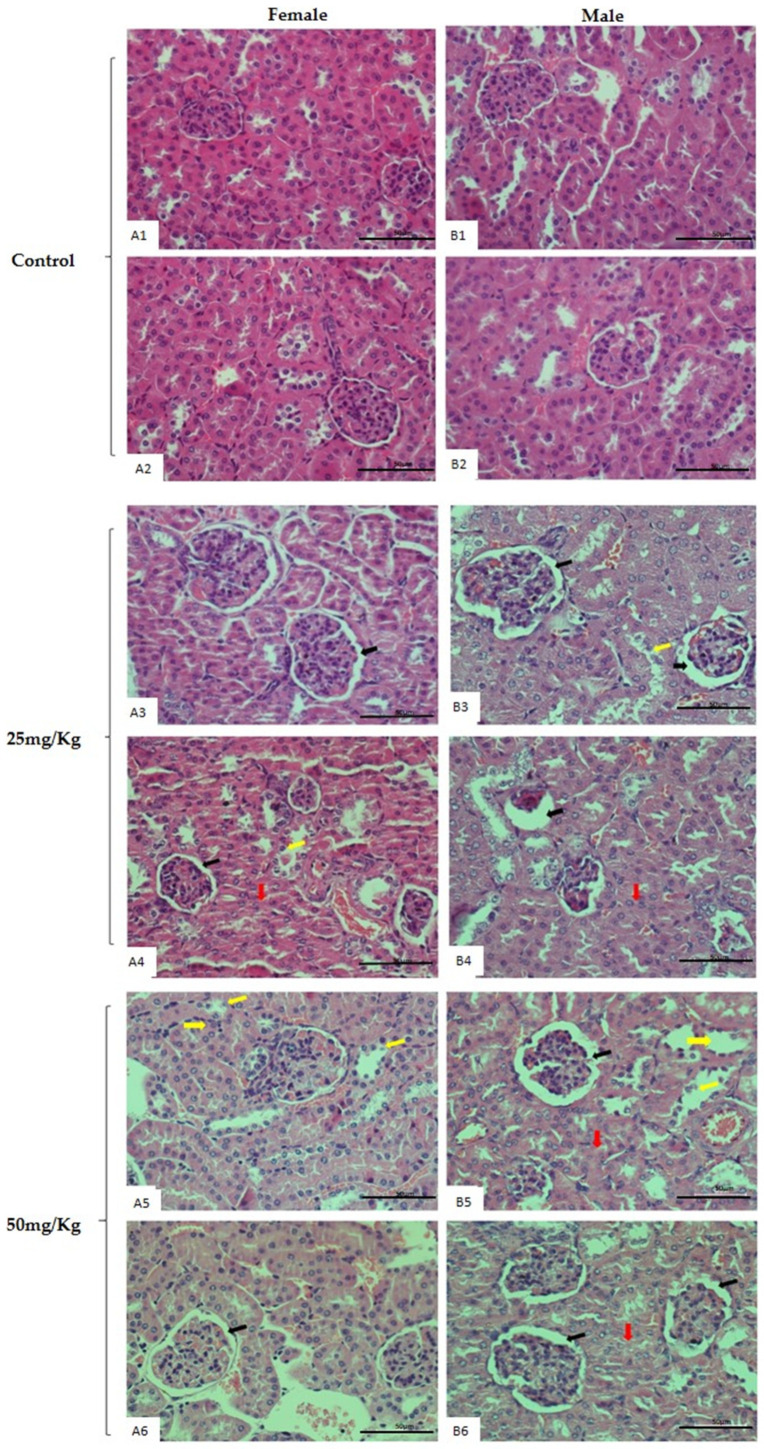
Representative histological images of kidney slices of male and female mice after 28-day repeated-dose toxicity study. Figures represent female (**A1**,**A2**) and male (**B1**,**B2**) control groups with normal histological aspects of renal tissue. Both female (**A3**,**A4**) and male mice (**B3**,**B4**) treated with 25 mg/kg of V 15 demonstrated vacuolization, cell peeling, cytoplasmic debris (**B3**,**A4**) (yellow arrows), focal tubular damage (**A4**,**B4**) (red arrows), and thickening of the basal membrane (**A3**,**B3**,**A4**,**B4**) (black arrows). The female (**A5**,**A6**) and male mice (**B5**,**B6**) treated with 50 mg/kg of V 15. (**A5**,**B5**) presents vacuolization, cytoplasmic debris (yellow arrows), and (**A6**,**B5**,**B6**) thickening of the basal membrane (black arrows) (H&E x40).

**Figure 5:**
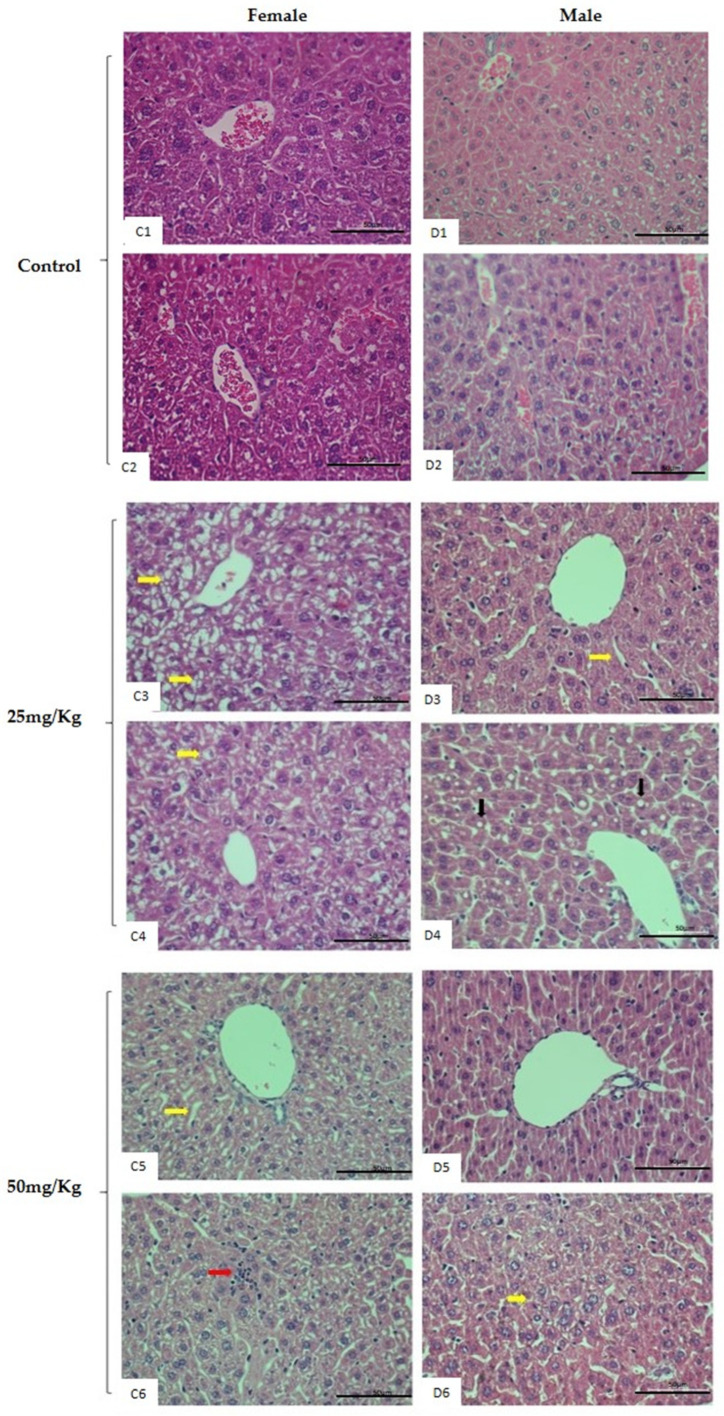
Representative histological images of liver slices of male and female mice after 28-day repeated-dose toxicity study. Figures represent (**C1**,**C2**) female and (**D1**,**D2**) male control groups with normal histological aspects of liver tissue. Both female (**C3**,**C4**) and (**D3**,**D4**) male mice treated with 25 mg/kg of V 15 showed (**C3**,**C4**,**D3**) focal degeneration, degeneration of hepatocytes (yellow arrows), and (**D4**) indications of local macrovesicular steatosis (black arrows). The female (**C5**,**C6**) and male animals (**D5**,**D6**) treated with 50 mg/kg of V 15, showing vacuolization (yellow arrow) (**C5**), (**D6**) hypertrophy of hepatocyte nuclei, and (**C6**) inflammation with mononuclear cell infiltrates (red arrow). (H&E x40) The scale bar used was 100 mm.

Alterations in mice liver and kidneys after treatment were confirmed by hematoxylin and eosin (H&E) histology analysis in V_15–25_ and V_15–50_ groups, as well as by comparison to the untreated mice group. The histopathological findings shown in Figure 4 and Figure 5 include vacuolization, mononuclear cell infiltrates, local macrovesicular steatosis, hypertrophy of hepatocyte nuclei, focal degeneration, hepatocyte degeneration, structural architecture damage, and hepatic remodeling in male and female mice treated with both doses. A microscopic evaluation of kidney tissue sections revealed focal tubular damage, swelling in the renal glomerulus, thickening of basement membranes, cell peeling, vacuolization, and cytoplasmic debris in male and female mice treated with both doses. Control animals of both sexes exhibited normal hepatic and kidney architecture.

## 3. Discussion

Polyoxometalates, including POVs, have been studied for potential biomedical applications, including antibacterial, antidiabetic, antiprotozoal, antiviral, and anticancer applications [1,2,3]. Given the wide structural and electronic range of POVs, a wide range of biological activities is apparent [46,47,48,49,50,51,52,53,54], and it is important to evaluate the possible toxicological effects. In general, short-term toxicity of vanadium administration has been observed in several species, including mammals [35,55,56] and fish [57,58]. However, the oral treatment by a single dose of a POV, and particularly a multivalent POV, has not been reported previously. The present study is the first report of an oral dose of POV repeated for 28 days in mice. These studies are important because some of the POVs have been found to have undesirable side effects [33], whereas others have been found to have beneficial effects [1,2,3]. The detailed mechanism of action of most POVs, however, is not understood and likely will vary depending on the specific POV or POM. We recently reported that V_15_ was able to activate signal transduction [9,13] and suggested that the redox properties of V_15_ were important to that mode of action. V_15_ has also been found to be effective in inhibiting cell growth [31], and the mode of action involved bacterial excretion of a temperature-insensitive material. In this case, V_15_ behaves similar to V_10_, which has several activities that are not attributed to its redox properties [1,22].

Several methods are useful to predict the toxicity of different types of substances [59,60], and the toxicity of the V_15_ cluster was screened against PMBC viability and a brine shrimp lethality bioassay [61,62]. Cell viability is one of the vital methods for toxicology analysis, which measures the cellular response to a toxic agent and provides information on survival and cell death. In this study, we found that V_15_ has a high cytotoxicity effect with low IC_50_ values in both PBMCs (17.5 μmol L^−1^) and the brine shrimp assay (17.9 mg mL^−1^). The studies demonstrated that molecules with IC_50_ values less than 100 μmol L^−1^ are considered very toxic [63]. Thus, comparable studies have shown the cytotoxicity of the vanadium compounds investigated [64,65,66]. Although the mechanism of vanadium toxicity remains to be elucidated and likely to vary depending on the compound examined, the cytotoxicity of vanadium is associated with the ability to accumulate vanadium ions and generate redox imbalance through an increase in intracellular ROS species [39].

The in vivo acute oral toxicity test represents a useful initial step in the toxicity assessment of compounds with potential biomedical applications [45]. Though the oral acute single administration of V_15_ did not cause any mortality, it showed a potential toxic effect through transitory behavior alterations, such as piloerection and lethargy after dosing, organ coefficients in the lung and liver, and biochemical parameters that indicated metabolic damage. Importantly, the toxicity evaluation is essential to identify a dose range and, hence, differences between efficacy and toxicity. This information would be useful to assess potential biomedical applications of a compound and identify any potential clinical symptoms that might be triggered by toxic levels of vanadium compounds under investigation.

In addition, there are no previous reports concerning repeated 28-day oral administration and toxicity of POVs. The Kaplan–Meier curve demonstrated that deaths was dose- and time-dependent. Survival analysis is important when the time between exposure and events is of clinical importance. Animal deaths occurred in both male and female Swiss mice sooner at higher dose treatments. Our data from the 28-day repeated-dose toxicity evaluation showed that male and female albino Swiss mice responded to the repeated exposure to V_15_ with observable side effects. All animals treated with 300 mg/kg of V_15_ died before the 12th day of the experiment (Figure 2). All of them demonstrated mortality/moribundity and clinical signs of toxicity, including lethargy, diarrhea, slimming, and abnormal gait and breathing.

Animals treated with a lower dose of 50 mg/kg of V_15,_ on the other hand, showed only a slight reduction in food and water intake. In addition, the weight of the kidney and testis was reduced in males treated with 50 mg/kg, and the weight of the skeletal muscles and reproductive organs was reduced in females treated with 25 and 50 mg/kg treatments. The observed reduction in organ weight could indicate that kidneys, skeletal muscles, and reproductive organs are potential targets following oral and repetitive treatments with soluble vanadium compounds. Kamboj and Kar showed that after two days of a single dose of vanadium exposure (0.08 nmol/kg), necrosis and a reduction in testes weight was detected in rats [67]. Furthermore, CD-1 male mice exposed to V_2_O_5_ inhalation for 12 weeks exhibited changes in the testes weight and the action of the testicular cytoskeleton, which might at least in part explain the reported reprotoxic effect of some vanadium compounds [68,69]. However, there are only a few reports concerning the effects of vanadium on the female reproductive system [70,71]. It has been shown that vanadium reduces glycogen content in the ovaries and uterus tissue, as well as protein content and acid phosphatase activity in the uterus. Furthermore, a microscopic evaluation of the ovaries revealed a decreasing mature follicle diameter, ovum disintegration, disorganized and hypertrophied growing follicles, and loose and fibrotic stroma [72]. Our results suggest that reprotoxic effects induced by POV are an important parameter that should be further investigated in future investigations of potential compounds for clinical use.

Hematological parameter evaluations were used to determine the extent of the harmful effect of V_15_ exposure. Repetitive dosing of V_15_ for 28 days at both doses caused similar toxic effects in male and female subjects. The impact of vanadium compounds on blood parameters has been demonstrated previously [73,74,75]. Sanchez-Gonzaléz et al. reported anemia in healthy rats treated with bis(maltolate)oxidovanadium(IV) dissolved in drinking water [73]. However, other studies have reported unchanged hematological parameters with treatment by different vanadium compounds. Specifically, Dai et al. (1994) showed that bis(maltolato)oxidovanadium(IV) (BMOV), ammonium metavanadate, and vanadyl sulfate in drinking water caused no hematological damage for 12 weeks [74]. Our results suggest a moderate normocytic normochromic anemia in both male and female mice treated with V_15_. Some vanadium compounds could affect physiological iron concentration [73] and may explain the anemia observed in animals exposed to POV. Furthermore, the thrombocytosis detected in V_15_-treated male and female mice may be due to inflammation, which is supported by the observed leukocyte count results. Our results agree with previous reports [75]. A higher predictive value for human toxicity for the hematological system is obtained upon the extrapolation of animal data. Thus, we suggest that V_15_ may be severely toxic to hematopoiesis.

An imbalance in oxidative stress is recognized as one potential cause of vanadium toxicity [76]. In addition, the great concern is the accumulation of vanadium in organs, such as the kidney or liver, which induces oxidative harm, lipid peroxidation, and toxicity [76,77,78,79]. Studies have demonstrated that some vanadium compounds may induce and exacerbate lipid peroxidation (LPO) and also affect human health. On the other hand, other vanadium compounds have protective effects on LPO [20,39]. Furthermore, vanadium compounds can sometimes generate free radicals, and thus form ROS/RNS directly, but they can also act indirectly through the effects on LPO [20,80,81,82]. Oster et al. (1993) showed that some vanadium compound treatments are correlated with TBAR production in both the liver and kidney in male rats [83]. Furthermore, Gândara et al. (2005) showed that decavanadate did not induce short-term LPO (12 h), but it later (24 h) increased LPO in the liver of *H. didactylus* [84]. Apart from this, rabbits exposed to vanadium pentoxide (V_2_O_5_) had increased LPO in the liver and kidney [85]. Our findings demonstrated an increase in LPO levels and a subsequent significant increase in liver lipid peroxidation in treated male animals at both doses and females at a higher dose of V_15_.

The prooxidant and antioxidant response induced by V_15_ was different in male and female mice. SOD activity was depressed in male mice, whereas it was stimulated in female mice treated with POV. In addition, the activity of the GSH antioxidant system decreased, whereas CAT activity increased only in female mice. Our data suggest that V_15_ has a higher toxic effect in female mice compared with male mice. GSH is the largest endogenous cellular redox buffer, and thus the most significant intracellular non-enzymatic antioxidant source [86]. Therefore, it could be possible that the GSH depletion and changes in the GSH:GSSH ratio may represent the mechanism of oxidative intensity and a higher toxicity of V_15_ in female mice. Female mice appear to be less vulnerable to oxidative stress under physiological conditions, but during stressful conditions, it is unclear whether female mice are more protected against oxidative stress than males [87]. The literature is controversial regarding the effects of vanadium compounds on GSH levels. NaVO_3_ in drinking water decreased GSH content in female rats [84]; on the other hand, ammonium metavanadate in drinking water increased GSH in the liver in the same animal model [88]. In 1993, Thompson and McNeill did not detect any noticeable differences in the amount of GSH in male rat livers treated with VOSO_4_ in drinking water for 12 weeks [89]. Some of these differences have been traced to specific conditions of the interaction of the vanadium compound with the GSH [90]. In conclusion, our results demonstrate that oxidative stress is associated with the toxicity of POVs, and that multiple mechanisms are likely responsible for V_15_ oral toxicity. According to our results, decavanadate inhibits respiration and induces membrane depolarization of the mitochondria, acting as a strong inhibitor, most likely through interference with the respiratory chain, and specifically complex III of the mitochondrial inner membrane, in the heart and liver [19,21].

The assessment of kidney and liver function in response to a new drug has been supported by the measurements of serum marker enzymes and/or metabolites [91,92]. In this study, the exposure of Swiss mice (male and female) to repeated doses of V_15_ produced several treatment-related effects, particularly in the groups treated with high doses of V_15_. Serum levels of ALT and AST have been observed as markers of liver injury, due to a variety of etiologies, such as medication toxicity and viral hepatitis [92]. In addition, a serum increase in ALT and AST levels is indicative of cell damage and a loss of the functional integrity of the hepatocyte membrane. Our data are comparable to previous reports on the liver toxicity of vanadium compounds in fish [57,84], broilers [93], and mammals [82,94,95]. These findings are related to an increase in liver histopathology with focal inflammation, diffused swelling of hepatocytes, and marked centrilobular hepatocellular injury. Pathological changes resulted from POV-induced free radical generation and the diminished antioxidant status of the compound. Similar histological alterations in the liver and kidney were seen in rabbits treated with vanadium pentoxide (V_2_O_5_) [85]. In addition, mice exposed to V_2_O_5_ inhalation for 6 weeks demonstrated inflammatory foci in liver parenchyma [95,96]. Cardiac myocytes also showed vacuolization and granular degeneration after 44 days of metavanadate (NH_4_VO_3_) exposure [97]. Taken together, previous results and observations in this paper suggest that oxidative stress is at the core of this histomorphology alteration and impairment of liver function and liver toxicity.

The kidney is a common target organ for toxicity and injury because of its exposure to drugs, including metallodrugs. Thus, renal function is usually assessed by serum levels of creatinine, uric acid, and BUN. These parameters may provide information on the effects of V_15_ on the tubular and/or glomerulus part of the kidney. V_15_ treatment increased serum creatinine levels in male mice at higher doses, which suggests the nephrotoxicity of this POV. In addition, hyperuricemia (HUA) may result from an imbalance in UA metabolism, which can lead to hyperuricemia and represent a very important signal of kidney toxicity. Both a reduction in glomerular filtration rate and an increase in net tubular absorption may result in HUA [91]. The kidney toxicity induced by vanadium compounds detected by an increasing UC has been demonstrated previously [94]. In this study, the increasing UA exerted by V_15_ plays an important role in inducing renal damage in both male and female mice. In addition, the microscopic appearance of kidney tissues of male and female mice dosed at 25 and 50 mg/kg of V_15_ showed that morphological alterations are compatible with nephrotoxicity induced by metals. Similar alterations were found in *H. didatylys* exposed to decavanadate oligomers [58] or ammonium metavanadate in broilers [93]. It has been demonstrated that vanadium may induce morphological damage to several organs, including kidneys. CD-1 mice exposed to vanadium oxide inhalation showed histological evidence of inflammatory foci [78]. Another study showed that V_10_ and a high-fat diet in Wistar rats caused harm to kidney cytoarchitecture [98]. Furthermore, vanadium compounds have the capacity to disturb the cell redox balance, which can result in damage to the antioxidant enzymatic and non-enzymatic systems. In general, these pathological changes in the liver and kidney resulted from POV-induced free radical generation and compromised the activity of the antioxidant status in these tissues.

## 4. Materials and Methods

### 4.1. Ethical Statement

The experimental strategy for these studies was approved by the human ethics committee of the Health Sciences Center/UFPE with the process number CAAE 04633218.6.0000.8807. Additionally, all animal care procedures were approved by the National Institutes of Health Guide for the Care and Use of Laboratory Animals (NIH Publication 8023, revised 1978) and followed the Ethics Committee on the Use of Animals of the Universidade Federal de Pernambuco (CEUA/UFPE, protocol 0021/2021).

### 4.2. Chemicals and General Methods

Ammonium metavanadate (NH_4_VO_3_, 99%) and D-mannitol (C_6_H_14_O_6_, 98%), both from Sigma-Aldrich^®^ (St. Louis, MO, USA), and tetramethylammonium chloride (C_4_H_12_ClN, 98%) from Fluka^®^ were used without purification. Deuterium oxide (D_2_O, 99.9 atom %D) was purchased from Oakwood Chemical and used as received. Ultrapure water (18.2 mΩ·cm) was used in the synthesis and characterization of V_15_, excepted when otherwise stated. The ^51^V NMR data were collected on a Bruker NEO 400 MHz spectrometer with a 105.2 MHz frequency for vanadium at 298 K, using 4096 scans in the f1 domain, 8 steady-state transitions, a spectra width of 800 ppm, a transmitter frequency offset of −548 ppm, 0.01 s relaxation delay, a 0.08192 s acquisition time, and a 16 μs pulse. Fourier-transform infrared (IR) spectra (400 to 4000 cm^–1^) were recorded from KBr pellets on a Bruker VERTEX-70 spectrometer with a resolution of 4 cm^−1^. Electron paramagnetic resonance in X-band (EPR) spectra (9.5 GHz) were recorded at 77 K on an X-band (9.5 GHz) Bruker EMX-Micro spectrometer using a pulverized sample. Electronic absorption spectra of 0.250 mmol L^−1^ aqueous solution of V_15_ were observed from 340 to 900 nm, using an AvaLight UV-Vis/NIR light source and an AvaSpex-UL S2048 Fiber-Optic Spectrometer.

#### 4.2.1. Synthesis of V_15_-(Me_4_N)_6_[V_15_O_36_(Cl)]

Mannitol (1.428 g, 7.839 mmol), tetramethylammonium chloride (0.7530 g, 6.870 mmol), and ammonium metavanadate (1.621 g, 13,86 mmol) were weighed and transferred to a round-bottom flask. Water (75 mL) was added, and the reaction mixture was stirred under reflux (which is 93 °C in Colorado) for 24 h. The solution changed from colorless to deep green in 2 h. A small amount of black powder started forming after 30 min. After 24 h, the reaction mixture was filtered to remove the black solid and kept at 4 °C to crystalize. Due to the small amount of black solid isolated, the insoluble black side product was confirmed by IR as (NH_4_)_2_V_3_O_8_ [29]. Pure V_15_ was obtained from the mother liquor after 4 days as dark green crystals, which were filtered off, washed with 10 mL of cold water, and dried under air. The average yield was 1.36 g, 81%, and the synthesis was reproduced 16 times to generate 10.864 g of V_15_. The dark-green crystals of V_15_ were insoluble in common organic solvents and soluble in hot water.

#### 4.2.2. Characterization of V_15_ in the Solid and Solution States

The solid V_15_, with a formula [(CH_3_)_4_N]_6_[V^IV^_8_V^V^_7_O_36_(Cl)], was confirmed by single-crystal X-ray diffraction analysis. One dark-green crystal of **V_15_** was subjected to analysis at 300 K on a Bruker D8 Venture diffractometer equipped with a Photon 100 CMOS detector using Mo-Kα radiation (μ(Mo-K_α_) = 0.711 mm^−1^). *Crystal data:* crystal system hexagonal *P*6_3/*mmc*_, a = b = 13.720 Å, c = 20.020 Å, α = β = 90°, and γ = 120°. The X-ray powder diffraction (PXRD) pattern of the V_15_ was registered at 40 kV and 30 mA on a Shimadzu XRD600 diffractometer equipped with a Cu-target tube (Cu-K_α_, k = 1.5418 Å). The calculated diffractogram of V_15_ was generated from the single-crystal crystallographic information (CIF) file available at Cambridge Crystallographic Data Centre, CCDC code 794586, using Mercury 4.0 software [99].

To verify the purity of the compound in bulk solid, powder X-ray diffraction pattern of V_15_ was compared with the previously described single-crystal X-ray diffraction structure, showing good correspondence.

The IR spectrum of solid V_15_ contains characteristic bands of the polyoxoanion at 560, 658, 728, and 793 cm^−1^ assigned to ν_as_(V−O−V) and 980 cm^−1^ to ν(V=O). Me_4_N^+^ bands appeared at 1288 cm^−1^ as ρ_r_(CH_3_), strong δ_as_, and partial reduction in the vanadium (V) and is compatible with the intra- and intermolecular exchange interaction among vanadium (IV) centers reported in the [V^IV^_8_V^V^_7_O_36_(Cl)]^6−^ (V_15_) polyoxoanion [29,43]. The EPR spectrum of the pulverized V_15_ sample was measured at 77 K and found to be like those reported previously [29].

The electronic absorption spectra were recorded for a 0.025 mmol L^−1^ aqueous solution of V_15_ and showed a ligand-to-metal charge transfer (LMCT, p(O)→d(V)) band below 400 nm and a broad band that extended from 600 nm to the near-infrared assigned to an intervalence charge transfer transition (IVCT, V^IV^ → V^V^) in the polyoxoanion. The spectra changed little over a period of 24 h, like studies by ^51^V NMR and EPR spectroscopy that monitored up to 34 h of bioassays with pH 7.4 [9,30]. These studies show that V_15_ slowly decomposes in aqueous solution to give V_15_ in the presence of species of oxovanadates (H_2_VO_4_^−^, H_2_V_2_O_7_^2−^, V_4_O_12_^4−^, V_5_O_15_^5−^) and the oxidovanadium(IV) complex [VO_2_(OH_2_)_4_]^+^. Importantly, no precipitation of black solid (NH_4_)_2_V_3_O_8_ was observed, further consistent with the lack of formation of the fresnoite type oxide (NH_4_)_2_VO_3_ and a solution containing hydrolytically stable V_15_.

### 4.3. Peripheral Blood Mononuclear Cells (PBMC) and Cytotoxicity Assay by 3-(4,5-Dimethyl-2-thiazolyl)-2,5-diphenyl-2H-tetrazolium bromide (MTT) of V_15_

PBMCs were obtained from the peripheral blood of healthy volunteers and collected in heparinized tubes. For PBMC separation, the peripheral blood was centrifuged using Ficoll Paque Plus (GE Healthcare Biosciences^®^, Pittsburgh, PA, USA). After separation, the cells were counted and placed in 96-well plates (5 × 10^5^ cells/100 μL/well) in RPMI-1640 medium (Gibco, ThermoFischer scientific^®^, Waltham, MA, USA), supplemented with L-Glutamine, 10% fetal bovine serum (FBS) (Gibco, ThermoFischer scientific^®^), 10 mmol L^−1^ HEPES (4-(2-hydroxyethyl)-1-piperazineethanesulfonic acid) (Gibco, Thermo Fischer scientific^®^), and 200 U/mL penicillin/streptomycin (Gibco). After being isolated, cells were exposed to V_15_ at serial concentrations of 10, 25, 50, and 100 µmol L^−1^ and then kept for 48 h in a humid atmosphere containing 5% CO_2_ at 37 °C. Cells treated with the solvent dimethyl sulfoxide (DMSO 0.1% *v*/*v*) were used as a control. The cytotoxicity was then quantified by the reduction of 3-(4,5-dimethyl-2-thiazolyl)-2,5-diphenyl-2H-tetrazolium bromide (MTT) to the purple reduction product formed in living cells. The absorbance was measured at 570 nm by the Elx808 (Biotek, Shoreline, WA, USA, USA apparatus^®^). Thus, the cytotoxic activity of V_15_ was quantified as the observed percentage of the control absorbance. The results are reported as the average value calculated from three separate experiments.

### 4.4. Toxicity against Larvae of Artemia Salina Leach

*Artemia salina* (Leach, 1819) (Tropical^®^, Rio de Janeiro, Brazil) dry cysts were used to evaluate V_15_-(Me_4_N)_6_[V_15_O_36_Cl] toxicity. Firstly, cysts were hydrated in artificial seawater (40 g sea salt/L, pH 8,0) and exposed to constant aeration at ambient temperature (25 ± 3 °C) for 48 h. After hatching, the larvae (nauplii) were transferred into vials (n = 10 per vial) containing seawater with 5, 10, 25, 50, 100, 125, 250, 500, and 1000 mg mL^−1^ final concentrations of V_15_ taken from the stock solutions of 1 mg mL^−1^ in DMSO 5% for 24 h at 25 ± 3 °C [61]. The control wells contained 10 nauplii in artificial seawater and DMSO 5%. Two experiments were performed in triplicate, with a total of 110 specimens per treatment, and assessments of mortality and survival of nauplii were carried out by observation of mobility for live nauplii identified after 24 h under a microscope.

### 4.5. Oral Acute Toxicity

The evaluation of acute oral toxicity was measured in accordance with Guideline 423, which describes the acute toxic class method reported by the Organization of Economic Co-operation and Development (OECD) for measuring acute oral toxicity of chemicals [45]. Nulliparous and non-pregnant female mice (three for each group) were acclimatized in a propylene cage for five days before dosage, fasted for 3 h, and weighed before the administration of a POV dose. The V_15_ was dissolved in NaCl 0.9% (*m*/*v*) and administered by gavage in mice in a single dose using animal feeding needles (100 μL/100 g b.w.). Animals were randomly divided into three groups with three animals each: (a) a control group treated with 0.9% (*m*/*v*) NaCl; (b) a low-dose group treated with 300 mg/kg of V_15_ (V_15–300_); and (c) a high-dose group treated with 2000 mg/kg of V_15_ (V_15–2000_).

All animals were closely monitored for the first four hours to identify piloerection as well as changes in eyes, skin, and fur, toxic effects on the mucous membranes, behavior pattern disorientation, asthenia, hypoactivity, hyperventilation, lethargy, lack of sleep, tremors, salivation, diarrhea, convulsion, coma, motor activity, or death. During the study, the animals were monitored daily for 14 days. Daily monitoring included measuring body weight, food intake, and water consumption. On the 14th day, the female mice were euthanized by intravenous injection of a solution containing both xylazine (80 mg/kg, i.p.) and ketamine (10 mg/kg, i.p.). The blood was drawn, and the organs collected for biochemical and macroscopic analysis. The LD_50_ was estimated based on mortality in each group as previously reported [100].

### 4.6. Toxicity Administered by the Repeated 28-Day Oral Toxicity Dose

The repeated oral dose 28-day toxicity test was carried out in accordance with Guideline 407 for rodents reported by the Organization of Economic Co-operation and Development (OECD) to evaluate chemicals for long-term oral toxicity [101]. Kaplan–Meier plots were used to assess the data on mice survival. The animals were acclimated in propylene cages for five days, at which point male, nulliparous, and non-pregnant female mice were fasted for three hours and weighed before they were given the compound dose. The mice were divided into groups of 10 (5 male and 5 female each) to be treated daily over 28 consecutive days by gavage using animal feeding needles (100 μL/100 g b.w.) The groups were divided as follows: (a) the control group was treated with 0.9% *m/v* NaCl; (b) the low-dose group was treated with 25 mg/kg of V_15_ (V_15–25_); (c) the high-dose group was treated with 50 mg/kg of V_15_ (V_15–50_); and (d) then treated with 300 mg/kg of V_15_ (V_15–300_). The number of deaths was recorded, and a Kaplan–Maier survival probability curve was plotted [102].

Animals were weighed once a week, and their basic morphological parameters, food and water intake, and any behavioral alterations were tracked. At the end of the 28 days of treatment, all animals were anesthetized by intraperitoneal administration of a xylazine and ketamine mixture (10 mg and 80 mg/kg, respectively) and euthanized by cervical dislocation. Blood samples were collected for biochemical and hematological examination, and the extracted liver and kidney organs were weighed and kept for histopathology.

### 4.7. Biochemical and Hematological Analysis

Mice fasted overnight were anesthetized as described above in the 14 days and 28 days following treatment. Blood samples were collected by the retro-orbital technique with or without heparin for hematological and serum biochemical analysis, respectively.

Hematological parameters included red blood cells (RBC), red cell volume distribution (RDW), hemoglobin concentration (HBG), hematocrit (HCT), platelet count (PLT), mean platelet volume (MPV), mean corpuscular volume (MCV), mean corpuscular hemoglobin (MCH), mean corpuscular hemoglobin concentration (MCHC), white blood cells (WBC), and lymphocytes (LYM). The test was performed using a multiparameter automatic hematology analyzer SDH-20, Labtest^®^, Curitiba, Brazil) designed for hematology testing samples.

The serum levels of several parameters were measured, including enzyme levels of alanine (ALT) and aspartate aminotransferase (AST), as well as metabolite levels including blood urea nitrogen (BUN), creatinine (CRE), total cholesterol (TC), total protein (TP), triacylglycerol (TG), and uric acid (UA) with a colorimetric assay using commercial kits (Lab Test Diagnostic SA^®^, Santa Lagoa, Brazil). Optical densities were measured by spectrophotometry using a Varioskan TM Lux multimode microplate reader on a Thermo Scientific^®^, Waltham, MA, USA instrument at the wavelengths designated on the datasheet for each biochemical parameter. Baseline measurements of controls were obtained by comparing the optical densities of the samples with the measurements using the appropriate standards provided in the kits. Data are showed in terms of U/mL (ALT and AST) and mg/dL for the others [16].

### 4.8. Oxidative Stress Evaluation in the Liver

Oxidative stress markers and lipid peroxidation in the liver were measured using thiobarbituric acid (TBAR), which measures the malondialdehyde (MDA) formed when lipids are oxidized according to the method reported previously [103]. Glutathione peroxidase (GPx) activity was measured in an assay using glutathione reductase as reported by Paglia and Valentine (1967) [104]; glutathione-reduced (GSH) levels were measured using a fluorometrical method as described by Hissin and Hilf (1976) [105]; the antioxidant activity of superoxide dismutase (SOD) was measured by monitoring superoxide anions generated by xanthine oxidase as described by Misra and Fridovich (1972) [106]; and the catalase (CAT) activity was monitored by measuring the decomposition of H_2_O_2_ by absorbance at 240 nm as reported by Aebi (1984) [107].

### 4.9. Organ Mass and Histopathological Analysis

After the 14th and 28th days of treatment, all mice were necropsied after blood withdrawal for anatomical localization and visible examination of detectable macroscopic organ changes (color, aspect, and size). Selected organs examined included the heart, liver, kidney, lungs, testicles, uterus, ovaries, spleen, adipose tissue, stomach, and soleus and extensor digitorum longus (EDL) muscles. These organs were carefully excised and trimmed of fat and connective tissue before weighing. The organ weight values were converted to g/100 g of body weight (percentage of body weight).

Following the 28-day treatment plan, the liver and kidney were fixed in 10% formalin buffer solution for 24 h at ambient temperature before being rinsed with water for four hours. They were then dehydrated stepwise using 70% (*v*/*v*) of EtOH. Dehydrated tissues were rendered transparent by addition of xylene. Tissues were added to pre-heated paraffin for sufficient infiltration, and after cooling, paraffin blocks were formed before being cut into 5 μm sections using a microtome (Leica^®^ RM 2025, Heidelberger, Germany). The paraffin was removed, and specimens were dehydrated and stained with hematoxylin-eosin for observation with an inverted optical microscope (Leica^®^, Heidelberger, Germany) connected to a video camera (Leica^®^ DFC 280, Wetzlar, Germany) and a computer monitor. An expert pathologist took pictures of the liver and kidney samples and examined them for any signs of cellular harm or morphological changes.

### 4.10. Statistical Analysis

Unless otherwise specified, the data were reported in triplicates as mean ± standard error of the mean (S.E.M). The data of V_15_-treated groups and their respective control groups was analyzed by one-way analysis of variance (ANOVA) followed by the Bonferroni test. The long-rank Kaplan–Meier survival test was used to compare the survival distribution of the different doses and treatment groups. A *p* value less than 0.05 was considered statistically significant. Graph Pad Prism^®^ (GraphPad Software, San Diego, CA, USA) version 6.0 software was used for all statistical analysis.

## 5. Conclusions

Administration of the mixed-valence polyoxovanadate (POV) V_15_, a nanosized anionic cluster, induces in vitro and in vivo toxicity in mice. The acute toxicity demonstrated that the LD_50_ value is more than 2000 mg/kg; thus, this POV has a low order of acute oral toxicity in mice and is a category 5 compound. However, the repeated 28-day assessment with systematic administration demonstrated toxic effects of this POV, especially in the liver and kidney tissues, and this is more toxic than in acute studies. The multivalent V_15_ was found to be stable under the condition resembling those used for the in vitro and in vivo studies. This suggests that the observed toxicity is mainly caused by the intact V_15_, considering the multivalent nature of the cluster and the ease of its oxidation. The redox properties of the cluster are likely related to its mode of action. Accordingly, the toxicity is attributed to redox damage produced by V_15_, which is consistent with induced hematological, hepatic, and renal toxicity observed in both male and female mice. Due to the oxidation of V_15_ and the resulting oxidative damage, tissues and metabolites are also likely involved in the process. Clinically, these results suggest that future research with any type of redox-active compounds may result in toxicity. Thus, there is a need to design non-toxic vanadium compounds, particularly POVs, for biomedical applications, which will be most beneficial if the delivery vehicle involved could control the reactivity of these compounds. These results should encourage researchers to develop new strategies to reduce the toxic effects of POVs, which could involve encapsulation in liposomes, other protective delivery systems, and complex-containing bioconjugate molecules.

## Figures and Tables

**Table 1:** Effects of the acute oral administration of V_15_ on total body weight (g), food intake (mL), and fluid intake (mL) after 14 days in different female mice groups.

Parameters	Control	V15–50	V15–300	V15–2000
Total body weight (g)	470.5 ± 11.7 ^a^	484.3 ± 15.8 ^a^	463.0 ± 14.8 ^a^	503.0 ± 20.0 ^a^
Total food intake (g)	96.5 ± 3.8 ^a^	89.7 ± 4.1 ^a^	102.7 ± 4.2 ^a^	103.3 ± 1.9 ^a^
Total fluid intake (mL)	194.0 ± 9.9 ^a^	189.3 ± 3.7 ^a^	234.0 ± 4.5 ^b^	235.0 ± 9.5 ^b^

The data are presented as the mean ± SEM (n = 3). The values with different superscript letters are statistically significant at *p* < 0.05 compared to control group, using one-way ANOVA.

**Table 2:** Effects of the acute oral administration of V_15_ on relative organ weight (mg/100 g of body weight) after 14 days in different female mice groups.

Organ	Control	V_15–50_	V_15–300_	V_15–2000_
Heart	0.56 ± 0.13 ^a^	0.69 ± 0.26 ^a^	0.54 ± 0.06 ^a^	0.57 ± 0.02 ^a^
Lung	0.54 ± 0.01 ^a^	0.58 ± 0.07 ^a^	0.72 ± 0.05 ^b^	0.68 ± 0.07 ^b^
Liver	5.93 ± 0.19 ^a^	5.15 ± 0.15 ^a^	5.94 ± 0.18 ^a^	6.70 ± 0.29 ^b^
Kidney	1.46 ± 0.04 ^a^	1.29 ± 0.03 ^a^	1.38 ± 0.036 ^a^	1.38 ± 0.04 ^a^
Spleen	0.54 ± 0.02 ^a^	0.46 ± 0.04 ^a^	0.64 ± 0.03 ^a^	0.66 ± 0.04 ^a^
Tibial anterior	0.13 ± 0.01 ^a^	0.15 ± 0.02 ^a^	0.12 ± 0.01 ^a^	0.13 ± 0.01 ^a^
Soleus	0.024 ± 0.002 ^a^	0.021 ± 0.001 ^a^	0.016 ± 0.002 ^a^	0.018 ± 0.001 ^a^

The data are presented as the mean ± SEM (n = 3). The values with different superscript letters are statistically significant at *p* < 0.05 compared to control group, using one-way ANOVA.

**Table 3:** Effects of the acute oral administration of V_15_ on serum biochemical profile after 14 days in different female mice groups.

Parameters	Control	V_15–50_	V_15–300_	V_15–2000_
AST (mmol/L)	58.5 ± 1.1 ^a^	87.0 ± 3.1 ^b^	117.9 ± 15.4 ^c^	139.7 ± 19.4 ^c^
ALT (mmol/L)	66.1 ± 5.3 ^a^	59.9 ± 0.6 ^a^	141.6 ± 8.9 ^b^	165.5 ± 14.4 ^b^
Total protein (mg/dL)	45.6 ± 4.2 ^a^	41.0 ± 1.1 ^a^	49.5 ± 2.5 ^a^	48.0 ± 3.6 ^a^
Albumin (μmol/L)	314.0 ± 29.0 ^a^	357.4 ± 4.8 ^a^	236.8 ± 11.3 ^b^	218.7 ± 5.4 ^c^
BUN (mg/dL)	11.2 ± 0.6 ^a^	11.2 ± 0.6 ^a^	11.2 ± 0.7 ^a^	10.5 ± 0.4 ^a^

The data are presented as the mean ± SEM (n = 5). Values with different superscript letters are statistically significant at *p* < 0.05 compared to control group, using one-way ANOVA. Abbreviations used are: ALT—alanine aminotransferase; AST—aspartate aminotransferase; BUN—blood urea nitrogen.

**Table 4:** Accumulative effects of the oral repeated administration of V_15_ on total body weight (g), food intake (g), and fluid intake (mL) in different male and female mice groups for 28-day toxicity evaluation.

Male			
Parameters	Control	V_15–25_	V_15–50_
Body weight (g)	1071.80 ± 21.8 ^a^	1.08000 ± 12.1 ^a^	1.07000 ± 29.2 ^a^
Food intake (g)	177.0 ± 6.6 ^a^	170.8 ± 2.8 ^a^	155.6 ± 5.4 ^b^
Fluid intake (mL)	316.0 ± 5.89 ^a^	327.5 ± 14.2 ^a^	308.40 ± 10.5 ^a^
**Female**			
**Parameters**	**Control**	**V_15–25_**	**V_15–50_**
Body weight (g)	941.20 ± 28.7 ^a^	928.80 ± 15.4 ^a^	912.70 ± 15.5 ^a^
Food intake (g)	146.8 ± 6.9 ^a^	154.2 ± 5.7 ^a^	136.3 ± 5.4 ^b^
Fluid intake (mL)	303.6 ± 9.0 ^a^	303.2 ± 18.1 ^a^	322.7 ± 3.5 ^a^

The data are presented as the mean ± SEM (n = 5). The values with different superscript letters are statistically significant at *p* < 0.05 compared to control group, using one-way ANOVA.

**Table 5:** Toxicity evaluation of the oral repeated administration of V_15_ on relative organ weight (mg/100 g of body weight) in different male and female mice groups after 28 days.

Male Mice			
Organ (mg/100 g of b.w.)	Control	V_15–25_	V_15–50_
Brain	1.13 ± 0.03 ^a^	1.10 ± 0.06 ^a^	1.05 ± 0.03 ^a^
Heart	0.41 ± 0.01 ^a^	0.51 ± 0.11 ^a^	0.45 ± 0.01 ^a^
Lung	0.62 ± 0.02 ^a^	0.63 ± 0.05 ^a^	0.52 ± 0.06 ^a^
Liver	4.69 ± 0.24 ^a^	5.41 ± 0.10 ^a^	4.99 ± 0.66 ^a^
Kidney	1.41 ± 0.05 ^a^	1.44 ± 0.08 ^a^	1.22 ± 0.08 ^b^
Spleen	0.35 ± 0.04 ^a^	0.42 ± 0.07 ^a^	0.39 ± 0.02 ^a^
Tibial anterior	0.15 ± 0.01 ^a^	0.14 ± 0.01 ^a^	0.13 ± 0.01 ^a^
Gastrocnemius	0.36 ± 0.01 ^a^	0.36 ± 0.02 ^a^	0.34 ± 0.02 ^a^
Teste	0.74 ± 0.08 ^a^	0.64 ± 0.08 ^b^	0.60 ± 0.01 ^b^
**Female mice**			
**Organ** **(mg/100 g of b.w.)**	**Control**	**V_15–25_**	**V_15–50_**
Brain	1.24 ± 0.09 ^a^	1.23 ± 0.04 ^a^	1.25 ± 0.03 ^a^
Heart	0.48 ± 0.04 ^a^	0.41 ± 0.01 ^a^	0.39 ± 0.02 ^a^
Lung	0.67 ± 0.09 ^a^	0.64 ± 0.05 ^a^	0.70 ± 0.05 ^a^
Liver	5.03 ± 0.22 ^a^	5.30 ± 0.23 ^a^	5.78 ± 0.05 ^a^
Kidney	1.34 ± 0.06 ^a^	1.16 ± 0.04 ^a^	1.14 ± 0.05 ^a^
Spleen	0.47 ± 0.04 ^a^	0.48 ± 0.04 ^a^	0.50 ± 0.05 ^a^
Tibial anterior	0.24 ± 0.06 ^a^	0.14 ± 0.01 ^b^	0.13 ± 0.01 ^b^
Gastrocnemius	0.25 ± 0.01 ^a^	0.28 ± 0.01 ^a^	0.31 ± 0.03 ^a^
Ovaries and uterus	1.32 ± 0.07 ^a^	0.94 ± 0.04 ^b^	0.92 ± 0.07 ^b^

The data are presented as the mean ± SEM (n = 5 for both male and female mice). Values with different superscript letters are statistically significant at *p* < 0.05 compared to control group, using one-way ANOVA.

**Table 6:** Evaluation of the effects of the repeated administration of V_15_ on hematological profile in different male and female mice groups after 28 days.

Male Mice			
Parameters	Control	V_15–25_	V_15–50_
RBC (×10^3^/μ)	6.80 ± 0.50 ^a^	6.90 ± 0.50 ^a^	5.50 ± 0.70 ^a^
Hemoglobin (%)	15.20 ± 1.30 ^a^	12.50 ± 0.50 ^b^	13.30 ± 0.60 ^b^
MCV (μm^3^)	58.50 ± 2.70 ^a^	46.70 ± 0.60 ^b^	47.70 ± 0.40 ^b^
MCH (pg)	20.60 ± 0.70 ^a^	18.70 ± 0.70 ^a^	17.40 ± 3.20 ^b^
MCHC (g/dL)	35.50 ± 1.80 ^a^	38.60 ± 0.10 ^a^	37.40 ± 6.80 ^a^
HCT (%)	43.40 ± 4.70 ^a^	32.20 ± 1.90 ^b^	26.40 ± 3.20 ^c^
Platelet count (×10^3^/μ)	531.00 ± 86.94 ^a^	778.00 ± 23.35 ^b^	942.75 ± 17.33 ^c^
WBC (×10^3^/μ)	14.00 ± 0.70 ^a^	13.30 ± 0.10 ^a^	14.50 ± 0.40 ^a^
Monocytes (%)	6.90 ± 1.00 ^a^	7.70 ± 0.30 ^a^	7.60 ± 2.50 ^a^
Lymphocyte (%)	89.50 ± 2.10 ^a^	89.00 ± 0.30 ^a^	87.40 ± 4.00 ^a^
**Female mice**			
**Parameters**	**Control**	**V_15–25_**	**V_15–50_**
RBC (×10^3^/μ)	14.80 ± 0.50 ^a^	13.90 ± 0.20 ^a^	15.2 ± 0.20 ^a^
Hemoglobin (%)	13.80 ± 0.60 ^a^	11.20 ± 0.80 ^b^	11.40 ± 0.70 ^b^
MCV (μm^3^)	56.80 ± 5.40 ^a^	47.30 ± 0.80 ^b^	42.40 ± 0.40 ^c^
MCH (pg)	20.90 ± 1.10 ^a^	15.10 ± 0.40 ^b^	15.30 ± 7.00 ^c^
MCHC (g/dL)	37.30 ± 1.60 ^a^	35.30 ± 2.40 ^a^	33.50 ± 17.50 ^a^
HCT (%)	37.30 ± 2.30 ^a^	31.80 ± 0.40 ^a^	11.4 ± 5.80 ^c^
Platelet count (×10^3^/μ)	438.82 ± 13.82 ^a^	499.00 ± 14.47 ^b^	911.66 ± 16.23 ^b^
WBC (×10^3^/μ)	6.70 ± 0.60 ^a^	7.40 ± 0.60 ^a^	3.60 ± 0.90 ^b^
Monocytes (%)	3.50 ±0.60 ^a^	4.90 ± 0.60 ^a^	6.10 ± 1.30 ^b^
Lymphocyte (%)	88.30 ± 4.70 ^a^	91.1 ± 0.60 ^a^	90.40 ± 2.40 ^a^

The data are presented as the mean ± SEM (n = 5). Values with different superscript letters are statistically significant at *p* < 0.05 compared to control group, using one-way ANOVA. Abbreviations used are: HCT—hematocrit; MCH—mean corpuscular hemoglobin; MCV—mean corpuscular volume; MCHC—mean corpuscular hemoglobin concentration; RBC—red blood cell; WBC—white blood cells.

**Table 7:** Effects of the oral administration of V_15_ on serum biochemical profile in different male and female mice groups after 28 days in repeated-dose toxicity evaluation.

Male Mice			
Parameters	Control	V_15–25_	V_15–50_
ALT (mmol/L)	39.90 ± 1.80 ^a^	103.70 ± 5.40 ^b^	122.50 ± 8.30 ^c^
AST (mmol/L)	74.80 ± 2.40 ^a^	81.90 ± 2.90 ^a^	145.40 ± 8.30 ^b^
Total protein (g/L)	46.60 ± 5.60 ^a^	46.00 ± 11.90 ^a^	51.60 ± 2.10 ^a^
BUN (mg/dL)	12.00 ± 0.20 ^a^	13.40 ± 0.90 ^a^	11.60 ± 0.01 ^a^
Creatinine (µmol/L)	29.80 ± 2.40 ^a^	23.86 ± 2.60 ^a^	71.90 ± 20.50 ^b^
Uric acid (µmol/L)	122.31 ± 15.30 ^a^	185.30 ± 17.30 ^b^	211.80 ± 32.70 ^b^
Total cholesterol (mmol/L)	1.60 ± 0.18 ^a^	1.58 ± 0.41 ^a^	1.80 ± 0.19 ^a^
Triglycerides (mmol/L)	1.62 ± 0.11 ^a^	1.84 ± 0.13 ^a^	1.30 ± 0.10 ^b^
**Female mice**			
**Parameters**	**Control**	**V_15–25_**	**V_15–50_**
ALT (mmol/L)	35.40 ± 2.70 ^a^	55.40 ± 3.10 ^b^	76.30 ± 12.10 ^c^
AST (mmol/L)	60.30 ± 3.10 ^a^	73.20 ± 4.70 ^a^	76.30 ± 12.10 ^b^
Total protein (g/L)	41.80 ± 5.40 ^a^	47.60 ± 6.00 ^a^	50.70 ± 1.50 ^a^
BUN (mg/dL)	11.49 ± 1.10 ^a^	13.41 ± 0.28 ^a^	11.74 ± 0.22 ^a^
Creatinine (µmol/L)	37.53 ± 6.44 ^a^	36.60 ± 3.60 ^a^	34.50 ± 1.80 ^a^
Uric acid (µmol/L)	114.92 ± 19.72 ^a^	170.60 ± 42.0 ^b^	204.10 ± 68.1 ^b^
Total cholesterol (mmol/L)	2.03 ± 0.14 ^a^	1.46 ± 0.17 ^b^	1.83 ± 0.06 ^a^
Triglycerides (mmol/L)	1.50 ± 0.14 ^a^	1.21 ± 0.12 ^b^	1.40 ± 0.07 ^a^

The data are presented as the mean ± SEM (n = 5). Values with different superscript letters are statistically significant at *p* < 0.05 compared to control group, using one-way ANOVA. Abbreviations used are: ALT—alanine aminotransferase; AST—aspartate aminotransferase; BUN—blood urea nitrogen.

**Table 8:** Effects of the repeated oral administration of V_15_ on oxidative stress parameters in different male and female mice groups and after 28 days.

Male Mice			
Parameters	Control	V_15–25_	V_15–50_
GSH (nmol/mg ptn)	10.2 ± 0.3 ^a^	10.4 ± 0.4 ^a^	10.1 ± 0.6 ^a^
GSSG (nmol/mg ptn)	7.4 ± 0.4 ^a^	7.4 ± 0.4 ^a^	7.3 ± 0.6 ^a^
GSH/GSSG (nmol/mg ptn)	1.4 ± 0.1 ^a^	1.4 ± 0.1 ^a^	1.4 ± 0.05 ^a^
MDA (U/mg ptn)	1.3 ± 0.1 ^a^	5.3 ± 0.3 ^b^	4.8 ± 0.5 ^b^
SOD (U/mg ptn)	19.9 ± 3.0 ^a^	14.0 ± 2.2 ^b^	15.7 ± 1.3 ^b^
CAT (U/mg ptn)	0.023 ± 0.005 ^a^	0.027 ± 0.010 ^a^	0.028 ± 0.010 ^a^
**Female mice**			
**Parameters**	**Control**	**V_15–25_**	**V_15–50_**
GSH (nmol/mg ptn)	6.6 ± 0.2 ^a^	7.2 ± 0.2 ^a^	6.9 ± 0.2 ^a^
GSSG (nmol/mg ptn)	2.4 ± 0.4 ^a^	5.0 ± 0.3 ^b^	4.2 ± 0.1 ^b^
GSH/GSSG (nmol/mg ptn)	2.8 ± 0.5 ^a^	1.5 ± 0.1 ^b^	1.6 ± 0.1 ^c^
MDA (U/mg ptn)	0.9 ± 0.1 ^a^	1.0 ± 0.2 ^a^	1.3 ± 0.1 ^b^
SOD (U/mg ptn)	16.6 ± 3.6 ^a^	33.7 ± 3.8 ^b^	24.3 ± 1.9 ^c^
CAT (U/mg ptn)	0.011 ± 0.003 ^a^	0.029 ± 0.008 ^b^	0.026 ± 0.004 ^b^

The data are presented as the mean ± SEM (n = 5). The values with different superscript letters are statistically significant at *p* < 0.05 compared to control group, using one-way ANOVA. Abbreviations used are: GSH—glutathione reduced; GSSG—glutathione oxidized; GSH/GSSG—ratio between GSH and GSSG; MDA—malondialdehyde; SOD—superoxide dismutase; CAT—catalase.

## Data Availability

All other data are available from the corresponding author upon reasonable request. Data is contained within the article or supplementary material.

## Supplementary Materials

The following supporting information can be downloaded from: https://www.mdpi.com/article/10.3390/ph16091232/s1, Figure S1: Comparison between simulated and experimental powder X-ray diffraction patterns of V_15_, in A. Crystal packing of [(CH_3_)_4_N]_6_[V_15_O_36_(Cl)] along with the c axis, in B. The weak C H···O interaction between the cation and the POV are shown in light blue; Figure S2: Infrared spectra recorded for products V_15_ (in black) and (NH_4_)_2_V_3_O_8_ (red) in KBr pellets; Figure S3: X-band EPR spectrum recorded for pulverized sample of V_15_ at 77 K; Figure S4: Electronic absorption spectra observed for a 0.025 mmol L^−1^ aqueous solution of V_15_ in t = 0 h, 20 min, 40 min, 1 h, 2 h, and 24 h.

## Abbreviations

ALB	Albumin
ALT	Alanine aminotransferase
ANOVA	One-way analysis of variance
AST	Aspartate aminotransferase
BMOV	Bis(maltolato)oxidovanadium(IV)
BUN	Blood urea nitrogen
b.w.	Body weight
CAT	Catalase
CEUA	Ethics Committee on the Use of Animals
CRE	Creatinine
CHO	Chinese Hamster Ovary Cells
DMSO	Dimethyl sulfoxide
DNA	Deoxyribonucleic acid
EDL	Extensor digitorum longus
EPR	Eletronic paragmagnetic ressonance
FBS	Fetal bovine serum
GSH	Glutathione reduced
GSSG	Glutathione oxidized
GLB	Globulin
GPx	Glutathione peroxidase
Hb	Hemoglobin
HCT	Hematocrit
HEPES	4-(2-hydroxyethyl)-1-piperazineethanesulfonic acid
i.p	Intraperitoneal administration
LC50	Lethal concentration median dose
LD50	Median lethal dose
LPO	Lipid peroxidation
LYM	Lymphocytes
MCH	Mean corpuscular hemoglobin
MCHC	Mean corpuscular hemoglobin concentration
MDA	Malondialdehyde
MVP	Mixed-valence polyoxovanadates
MTT	3-(4,5- dimethyl-2-thiazolyl)-2,5-diphenyl-2H-Tetrazolium bromide
NMR	Nuclear magnetic ressonance
o.g.	Oral gavage
OECD	Organization for Economic Cooperation and Development
PBMC	Peripheral blood mononuclear cells
POM	Polyoxometalate
POV	Polyoxovanadate
RBC	Red blood cells
RNS	Reactive nitrogen species
ROS	Reactive oxygen species
Rpm	Rotation per minute
SD	Standard deviation
SEM	Standard error of the mean
SOD	Superoxide dismutase
STZ	Streptozotocin
TC	Total cholesterol
TP	Total protein
TBAR	Thiobarbituric acid
UA	Uric acid
WBC	White blood cells
