# Supplementary material for: In Vitro, Oral Acute, and Repeated 28-Day Oral Dose Toxicity of a Mixed-Valence Polyoxovanadate Cluster
Source: Pharmaceuticals (Basel). 2023 Aug 30;16(9):1232. doi: 10.3390/ph16091232 (PMC10536805; doi:10.3390/ph16091232)
Supplement: Supplementary file 1 [file pharmaceuticals-16-01232-s001.zip › pharmaceuticals-2491426-supplementary.pdf]

Supplementary material

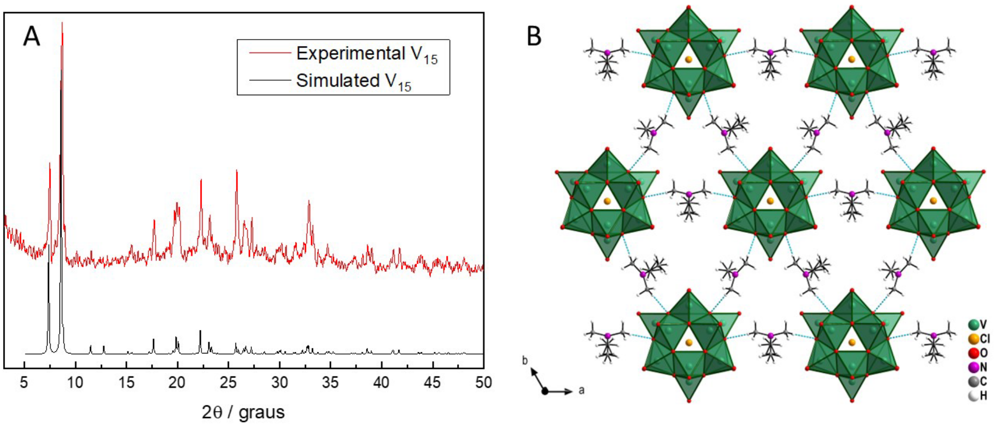

Figure S1. Comparison between simulated and experimental powder X-ray diffraction patterns of V_{15}, in A. Crystal packing of $[(CH_3)_4N]_6[V_{15}O_{36}(Cl)]$ along with the c axis, in B. The weak C–H···O interaction between the cation and the POV are showed in light blue.

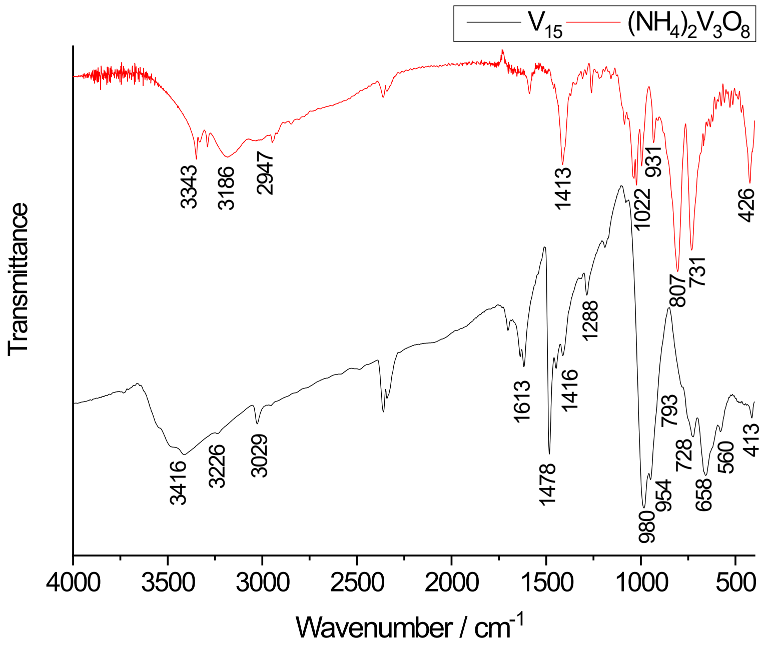

Figure S2. Infrared spectra recorded for products V_{15} (in black) and $(NH_4)_2V_3O_8$ (red) in KBr pellets.

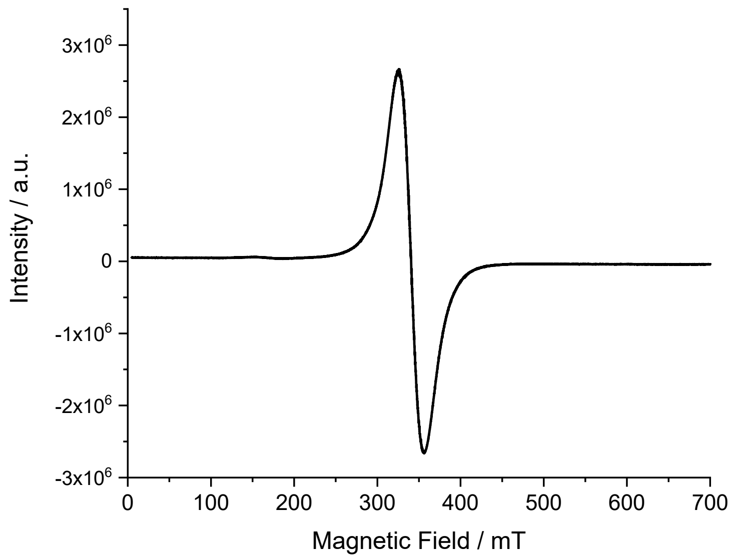

Figure S3. X-band EPR spectrum recorded for pulverized sample of V_{15} at 77 K.

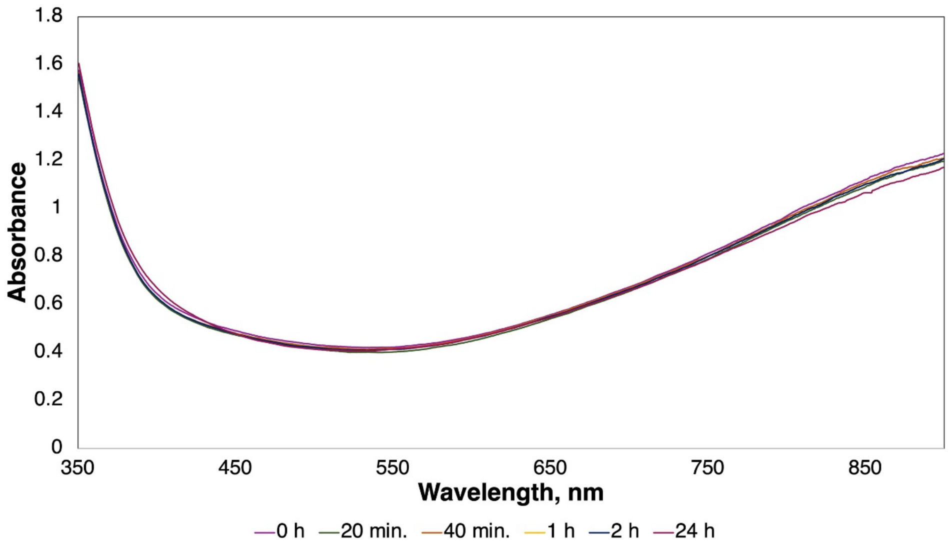

Figure S4. Electronic absorption spectra observed for a 0.025 mmol L^{-1} aqueous solution of V_{15} in t = 0 h, 20 min, 40 min, 1h, 2h, and 24h.
